# The transverse momentum spectrum of weak gauge bosons at N${}^3$LL + NNLO

**DOI:** 10.1140/epjc/s10052-019-7324-0

**Published:** 2019-10-22

**Authors:** Wojciech Bizoń, Aude Gehrmann-De Ridder, Thomas Gehrmann, Nigel Glover, Alexander Huss, Pier Francesco Monni, Emanuele Re, Luca Rottoli, Duncan M. Walker

**Affiliations:** 1 Institut für Theoretische Teilchenphysik (TTP), KIT, 76128 Karlsruhe, Germany; 2 Institut für Kernphysik (IKP), KIT, 76344 Eggenstein-Leopoldshafen, Germany; 3 Institute for Theoretical Physics, ETH, 8093 Zürich, Switzerland; 4 Department of Physics, University of Zürich, 8057 Zurich, Switzerland; 5 Institute for Particle Physics Phenomenology, Durham University, Durham, DH1 3LE UK; 6 Theoretical Physics Department, CERN, 1211, Geneva 23, Switzerland; 7 LAPTh, Université Grenoble Alpes, Université Savoie Mont Blanc, CNRS, 74940 Annecy, France; 8 Dipartimento di Fisica G. Occhialini, U2, Università degli Studi di Milano-Bicocca, Piazza della Scienza, 3, 20126 Milan, Italy; 9 INFN, Sezione di Milano-Bicocca, 20126 Milan, Italy

## Abstract

We present accurate QCD predictions for the transverse momentum ($p_\perp$) spectrum of electroweak gauge bosons at the LHC for $13~\mathrm {TeV}$ collisions, based on a consistent combination of a NNLO calculation at large $p_\perp$ and N$^3$LL resummation in the small $p_\perp$ limit. The inclusion of higher order corrections leads to substantial changes in the shape of the differential distributions, and the residual perturbative uncertainties are reduced to the few percent level across the whole transverse momentum spectrum. We examine the ratio of $p_\perp$ distributions in charged- and neutral-current Drell–Yan production, and study different prescriptions for the estimate of perturbative uncertainties that rely on different degrees of correlation between these processes. We observe an excellent stability of the ratios with respect to the perturbative order, indicating a strong correlation between the corresponding QCD corrections.

**Electronic supplementary material:**

The online version of this article (10.1140/epjc/s10052-019-7324-0) contains supplementary material, which is available to authorized users.

## Introduction

The differential spectrum of electroweak gauge bosons, measured via their leptonic decays, is among the most prominent observables at the LHC.

Owing to the outstanding precision of their experimental measurement [1–14], such observables allow for a precise extraction of some of the Standard Model (SM) parameters – such as the *W* boson mass [13], or parton densities [15–18] – as well as for the calibration of widely used event generators and analysis tools. For this reason, an accurate theoretical understanding of such observables is paramount to exploit the precise data and perform meticulous tests of the SM.

Inclusive and differential distributions for neutral and charged Drell–Yan (DY) production with lepton pair invariant mass *M* are nowadays known with very high precision. The total cross section is known fully differentially in the Born phase space up to NNLO [19–27], while differential distributions in transverse momentum $p_\perp$ were recently computed up to NNLO both for *Z*- [28–33] and *W*-boson [34–36] production. In the DY distributions, electroweak corrections become important especially at large transverse momenta, and they have been computed to NLO accuracy in [37–40].

In kinematical regimes dominated by soft and collinear radiation, the fixed-order perturbative series for the differential $p_\perp$ distribution is affected by large logarithmic terms of the form $\alpha _\mathrm {s}^n L^{2n-1}/p_\perp$, with $L\equiv \ln (M/p_\perp )$, which must be resummed to all orders for a reliable theoretical prediction. In such regimes, the perturbative (logarithmic) accuracy is defined in terms of the *logarithm* of the cumulative cross section $\mathrm{\Sigma }$ as

1 $$\begin{aligned} \ln \left( \mathrm{\Sigma }(p_\perp )\right)&\equiv \ln \left( \int _0^{p_\perp } \mathrm {d} p_\perp ' \; \frac{\mathrm {d} \mathrm{\Sigma }(p_\perp ')}{\mathrm {d} p_\perp '} \right) \nonumber \\&= \sum _n \left\{ \mathcal{O}\left( \alpha _\mathrm {s}^nL^{n+1}\right) + \mathcal{O}\left( \alpha _\mathrm {s}^nL^{n}\right) + \dots \right\} . \end{aligned}$$

One refers to the dominant terms $\alpha _\mathrm {s}^n L^{n+1}$ as leading logarithmic (LL), to terms $\alpha _\mathrm {s}^n L^{n}$ as next-to-leading logarithmic (NLL), to $\alpha _\mathrm {s}^n L^{n-1}$ as next-to-next-to-leading logarithmic (NNLL), and so on. The resummation of the $p_\perp$ spectrum of SM bosons has been studied in a multitude of theoretical formulations throughout the years [41–51], and the current state of the art for phenomenological studies at the LHC reaches N$^3$LL accuracy [51–54].

In this article, we reach a new milestone in the theoretical description of transverse momentum distributions in both neutral and charged DY production, aiming for percent level precision throughout the full kinematical range. This is achieved by matching the fixed-order NNLO QCD predictions with the N${}^3$LL resummation of large logarithmic corrections. We adopt the momentum-space formulation of Refs. [49, 51], in which the resummation is performed by generating the QCD radiation by means of a Monte Carlo (MC) algorithm. All the necessary ingredients for the N${}^3$LL $p_\perp$ resummation have been computed in Refs. [55–61]. The combined framework enables fully differential N${}^3$LL+NNLO predictions for distributions that take proper account of the fiducial volume definitions used in the experimental measurements.

The article is organised as follows. In Sect. 2 we briefly review the computation of the NNLO differential distributions in DY-pair production with the parton-level code NNLOjet, as well as the resummation for the $p_\perp$ distributions using the computer program RadISH. Section 3 describes our results for $13~\mathrm {TeV}$ LHC collisions. Finally, Sect. 4 contains our conclusions.

## Setup of the calculation

In this section we give a brief overview of the computational setup, and describe the ingredients of both the fixed order (Sect. 2.1) and the resummed (Sect. 2.2) calculations.

### Fixed order

For the calculation of the DY process, we consider the off-shell production of either a pair of charged leptons (mediated by both a *Z* boson and a virtual photon) or a charged lepton and a neutrino (mediated by $W^{\pm }$ bosons), in association with partonic jets. The jet requirement is replaced by a lower cut on the transverse momentum of the pair, that acts as an infrared regulator of the fixed-order calculation, hence preventing the radiation from being entirely unresolved.

The NNLO QCD predictions for neutral and charged DY production have been obtained in Refs. [28–36]. Relative to the LO distribution, in which the leptonic system recoils against a single parton, the NNLO calculation receives contributions from configurations with two extra partons (RR: double-real corrections [62–66]), with one extra parton and one extra loop (RV: real-virtual corrections [62, 63, 67–70]) and with no extra partons but two extra loops (VV: double-virtual corrections [71–74]). Each of the three contributions is separately infrared divergent either in an implicit manner from phase-space regions where the partonic radiation becomes unresolved (soft and/or collinear), or in a explicit manner from infrared poles in virtual loop corrections. Only the sum of the three contributions is finite.

We perform the calculation using the parton-level generator NNLOjet, which implements the antenna subtraction method [75–77] to isolate infrared singularities and to enable their cancellation between different contributions prior to the numerical phase-space integration. The NNLO calculation can be structured as

2 $$\begin{aligned} \sigma ^\text {NNLO}_{X+\text {jet}}&=\int _{\varPhi _{X+3}}\Big (\mathrm {d} \sigma ^{RR}_\text {NNLO}-\mathrm {d} \sigma ^S_\text {NNLO}\Big )\nonumber \\&+\int _{\varPhi _{X+2}}\Big (\mathrm {d} \sigma ^{RV}_\text {NNLO}-\mathrm {d} \sigma ^T_\text {NNLO}\Big )\nonumber \\&+\int _{\varPhi _{X+1}}\Big (\mathrm {d} \sigma ^{VV}_\text {NNLO}-\mathrm {d} \sigma ^U_\text {NNLO}\Big ). \end{aligned}$$

The antenna subtraction terms, $\mathrm {d} \sigma ^{S,T,U}_\text {NNLO}$, are constructed from antenna functions [75, 78–82] to cancel infrared singularities between the contributions of different parton multiplicities. The integrals are performed over the phase space ${\varPhi _{X+1,2,3}}$ corresponding to the production of the colour singlet in association with one, two or three partons in the final state. The integration over the final-state phase space is fully differential such that any infrared-safe observable $\mathcal O$ can be studied through differential distributions as $\mathrm {d} \sigma ^\text {NNLO}_{X+\text {jet}}/\mathrm {d} \mathcal O$.

The matching of the above NNLO prediction to a resummed calculation in the small $p_\perp$ limit is computationally very challenging. At small $p_\perp$, both the matrix elements and the subtraction terms grow rapidly in magnitude due to the presence of un-cancelled infrared singularities. This results in large numerical cancellations between them that ultimately challenge the stability of the final prediction. The finite remainder of such cancellations needs to be numerically stable in order to be consistently combined with a resummed calculation and extrapolated to the limit $p_\perp \rightarrow 0$. The stability of NNLOjet in this extreme regime has been tested thoroughly against the expansion of the N$^3$LL resummations in Refs. [52, 53], where it is shown that the NNLO calculation can be reliably obtained down to very small $p_\perp$ values.

The residual infrared (logarithmic) divergences that persist in the $p_\perp \rightarrow 0$ limit are cancelled by combining the fixed-order computation with a resummed calculation, where the logarithms in the fixed-order prediction are subtracted and replaced by the sum of the corresponding enhanced terms to all orders in perturbation theory. This procedure is discussed in the following Sect. 2.2.

### Resummation and matching

The resummation is performed in momentum space by means of the method formulated in Refs. [49, 51] and implemented in the computer code RadISH. In this approach, the factorisation properties of the QCD matrix elements in the soft and collinear limits are exploited to devise a numerical procedure to generate the radiation at all perturbative orders. This allows for the resummation of the large logarithmic terms in a fashion that is similar in spirit to a Monte Carlo generator. A detailed technical description of the method can be found in Refs. [49, 51], and the formulae up to N$^3$LL accuracy are collected in Ref. [53] (Sect. 3 and Appendix B).

In order to have a reliable prediction across the whole $p_\perp$ spectrum, the fixed-order and resummed predictions must be consistently combined through a matching procedure. The matching is performed in such a way that it reduces to the resummed calculation at small $p_\perp$, while reproducing the fixed-order prediction at large transverse momentum. At a given perturbative order, one can adopt various schemes that differ from one another by terms beyond the considered order. In the present analysis we adopt the multiplicative scheme formulated in Refs. [53, 83], in which the matching is performed at the level of the cumulative distribution (1) as follows:

3 $$\begin{aligned} \mathrm{\Sigma }_\mathrm{match}^\mathrm{N^3LL}(p_\perp ) = \frac{\mathrm{\Sigma }^\mathrm{N^3LL}(p_\perp )}{\mathrm{\Sigma }^\mathrm{N^3LL}_\mathrm{asym.} } \left[ \mathrm{\Sigma }^\mathrm{N^3LL}_\mathrm{asym.} \frac{\mathrm{\Sigma }^\mathrm{N^3LO}(p_\perp )}{\mathrm{\Sigma }_\mathrm{exp.}^\mathrm{N^3LL}(p_\perp )}\right] _\mathrm{N^3LO}, \end{aligned}$$

where $\mathrm{\Sigma }_\mathrm{exp.}^\mathrm{N^3LL}$ denotes the expansion of the resummation formula $\mathrm{\Sigma }^\mathrm{N^3LL}$ to $\mathcal{O}(\alpha _s^3)$ (N$^3$LO), and the whole squared bracket in Eq. (3) is expanded to N$^3$LO. It should be recalled that $\mathcal{O}(\alpha _s^3)$ corresponds to N$^3$LO in the total (i.e. $p_\perp$-integrated) cross section and in any cumulative distribution, while being NNLO in the fixed-order $p_\perp$-differential cross section.

In the above equation, $\mathrm{\Sigma }^\mathrm{N^3LO}$ is the cumulative fixed-order distribution at N$^3$LO, i.e.

4 $$\begin{aligned} \mathrm{\Sigma }^\mathrm{N^3LO}(p_\perp ) = \sigma _\mathrm{tot}^\mathrm{N^3LO} - \int _{p_\perp }^{\infty } \mathrm {d} p_\perp ' \; \frac{\mathrm {d} \mathrm{\Sigma }^\mathrm{NNLO}(p_\perp ')}{\mathrm {d} p_\perp '}, \end{aligned}$$

where $\sigma _\mathrm{tot}^\mathrm{N^3LO}$ is the total cross section for the charged or neutral DY processes at N$^3$LO, and $\mathrm {d} \mathrm{\Sigma }^\mathrm{NNLO}/\mathrm {d} p_\perp '$ denotes the corresponding NNLO $p_\perp$-differential distribution obtained with NNLOjet. Both of these quantities are accurate to $\mathcal{O}(\alpha _s^3)$. Since the N$^3$LO inclusive cross section for DY production is currently unknown, we approximate it with the NNLO cross section [19–27] in the following. This approximation impacts only terms at N$^4$LL order, and is thus beyond the accuracy considered in this study.

Finally, the quantity $\mathrm{\Sigma }^\mathrm{N^3LL}_\mathrm{asym.}$ is defined as the asymptotic ($p_\perp \gg M$) limit of the resummed cross section

5 $$\begin{aligned} \mathrm{\Sigma }^\mathrm{N^3LL}(p_\perp )\xrightarrow [p_\perp \gg M]{} \mathrm{\Sigma }^\mathrm{N^3LL}_\mathrm{asym.} . \end{aligned}$$

This prescription ensures that, in the $p_\perp \gg M$ limit, Eq. (3) reproduces by construction the fixed-order result, while in the $p_\perp \rightarrow 0$ limit it reduces to the resummed prediction. The detailed matching formulae can be found in Appendix A of Ref. [53].

In the next section, we will also report matched predictions at lower perturbative orders, NNLL + NLO and NLL + LO, that are obtained from the following matched cumulative distributions

6 $$\begin{aligned} \mathrm{\Sigma }_\mathrm{match}^\mathrm{NNLL}(p_\perp )&= \frac{\mathrm{\Sigma }^\mathrm{NNLL}(p_\perp )}{\mathrm{\Sigma }^\mathrm{NNLL}_\mathrm{asym.} } \left[ \mathrm{\Sigma }^\mathrm{NNLL}_\mathrm{asym.} \frac{\mathrm{\Sigma }^\mathrm{NNLO}(p_\perp )}{\mathrm{\Sigma }_\mathrm{exp.}^\mathrm{NNLL}(p_\perp )}\right] _\mathrm{NNLO}\,, \end{aligned}$$

7 $$\begin{aligned} \mathrm{\Sigma }_\mathrm{match}^\mathrm{NLL}(p_\perp )&= \frac{\mathrm{\Sigma }^\mathrm{NLL}(p_\perp )}{\mathrm{\Sigma }^\mathrm{NLL}_\mathrm{asym.} } \left[ \mathrm{\Sigma }^\mathrm{NLL}_\mathrm{asym.} \frac{\mathrm{\Sigma }^\mathrm{NLO}(p_\perp )}{\mathrm{\Sigma }_\mathrm{exp.}^\mathrm{NLL}(p_\perp )}\right] _\mathrm{NLO}\,. \end{aligned}$$

The above matching schemes guarantee that in the large-$p_\perp$ limit the matched cumulative cross sections reproduce, by construction, the following total cross sections

8 $$\begin{aligned} \mathrm{\Sigma }_\mathrm{match}^\mathrm{N^3LL}(p_\perp )&\xrightarrow [p_\perp \gg M]{} \sigma _\mathrm{tot}^\mathrm{NNLO}\,,\nonumber \\ \mathrm{\Sigma }_\mathrm{match}^\mathrm{NNLL}(p_\perp )&\xrightarrow [p_\perp \gg M]{} \sigma _\mathrm{tot}^\mathrm{NNLO}\,,\nonumber \\ \mathrm{\Sigma }_\mathrm{match}^\mathrm{NLL}(p_\perp )&\xrightarrow [p_\perp \gg M]{} \sigma _\mathrm{tot}^\mathrm{NLO}\,. \end{aligned}$$

We stress once more that the $\mathrm{\Sigma }_\mathrm{match}^\mathrm{N^3LL}$ reproduces the NNLO total cross section at large $p_\perp$ since the N$^3$LO result for the inclusive DY process is currently unknown. The nominal accuracy of the predictions is unaffected by this choice.

The final normalised distributions that will be reported in Sect. 3 are obtained by differentiating Eqs. (3), (6) and (7), and dividing by the respective total cross sections of the right hand side of Eq. (8).

We recall that the resummed calculation contains a Landau singularity arising from configurations where the radiation is generated with transverse momentum scales $k_\perp \sim M\, \exp \left\{ -1/(2\beta _0\alpha _s)\right\}$ (with $\alpha _s = \alpha _s(M)$ and $\beta _0 = (11\,C_A-2\,n_f)/(12\pi )$). In the predictions presented in the following, we set the results to zero when the hardest radiation’s transverse momentum reaches the singularity. For the leptonic invariant masses studied here, this procedure acts on radiation emitted at very small transverse momentum that, due to the vectorial nature of the observable [41, 51], gives a very small contribution to the spectrum. We however stress that for a precise description of this kinematic regime, a thorough study of the impact of non-perturbative corrections is necessary.

## Results at the LHC

In this section we report our numerical results for the neutral and charged DY transverse momentum distributions at N$^3$LL+NNLO.

We consider *pp* collisions at a centre-of-mass energy of $13~\mathrm {TeV}$, and we use the NNLO NNPDF3.1 parton distribution function set [15] with $\alpha _\mathrm {s}(M_Z) = 0.118$. The parton densities are evolved from a low scale $Q_0\sim 1~\mathrm {GeV}$ forwards with LHAPDF [84], which correctly implements the heavy quark thresholds in the PDFs. All convolutions are handled with the Hoppet package [85]. In the results reported below, we use the NNLO DGLAP evolution of the adopted PDF set for all perturbative orders shown in the figures. Although the NNLO corrections to the PDF evolution are formally of order N$^3$LL, we include them also in the NLL and NNLL predictions in order to guarantee a consistent treatment of the quark thresholds in the parton densities. We note that this choice will lead to numerical differences in comparison to other NLL and NNLL results shown in the literature.

We adopt the $G_\mu$ scheme with the electro-weak parameters taken from the PDG [86], that is

9 $$\begin{aligned}&M_Z = 91.1876~\mathrm {GeV} ,\quad M_W = 80.379~\mathrm {GeV} ,\nonumber \\&\mathrm{\Gamma }_Z = 2.4952~\mathrm {GeV} ,\quad \mathrm{\Gamma }_W = 2.085~\mathrm {GeV} ,\nonumber \\&G_F=1.1663787\times 10^{-5}~\mathrm {GeV} ^{-2}\,. \end{aligned}$$

Moreover, we set the CKM matrix equal to the identity matrix, and we have verified that this approximation is accurate at the few-permille level. For both neutral-current and charged-current DY we apply fiducial selection cuts that resemble the ones used by ATLAS in previous analyses [4].

The final state for the neutral DY production is defined by applying the following set of fiducial selection cuts on the leptonic pair:

10 $$\begin{aligned}&|\mathbf {p}_{\perp }^{\ell ^\pm }| > 25~\mathrm {GeV} ,\quad |\eta ^{\ell ^\pm }|< 2.5,\nonumber \\&~ 66~\mathrm {GeV}< \, M_{\ell \ell } \,< 116~\mathrm {GeV} , \end{aligned}$$

where $\mathbf {p}_{\perp }^{\ell ^\pm }$ are the transverse momenta of the two leptons, $\eta ^{\ell ^\pm }$ are their pseudo-rapidities in the hadronic centre-of-mass frame, and $M_{\ell \ell }$ is the invariant mass of the di-lepton system. The central factorisation and renormalisation scales are chosen to be $\mu _R = \mu _F = \sqrt{M_{\ell \ell } ^2 + |\mathbf {p}_\perp ^{Z}|^2 }$ and the central resummation scale is set to $Q=M_{\ell \ell }/2$.

In the case of charged DY production, the fiducial volume is defined as11where  is the missing transverse energy vector and12The central factorisation and renormalisation scales are chosen to be $\mu _R = \mu _F = \sqrt{M_{\ell \nu } ^2 + |\mathbf {p}_\perp ^{W}|^2 }$ and the central resummation scale is again set to $Q=M_{\ell \nu }/2$.

In both processes, we assess the missing higher-order uncertainties by performing a variation of the renormalisation and factorisation scales by a factor of two around their respective central values whilst keeping $1/2 \le \mu _R/\mu _F \le 2$. In addition, for central factorisation and renormalisation scales, we vary the resummation scale *Q* by a factor of two in either direction. The final uncertainty is built as the envelope of the resulting nine-scale variation.

**Fig. 1:**
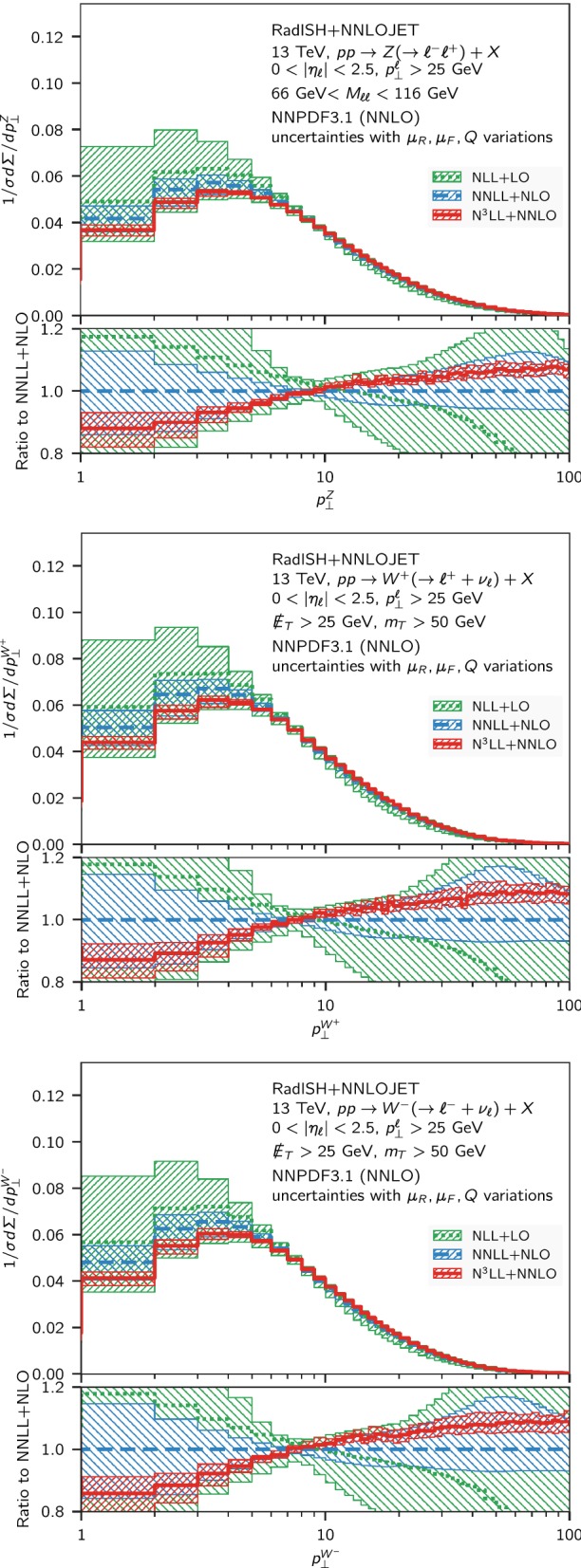
Comparison of the normalised transverse momentum distribution for neutral and charged Drell–Yan pair production at NLL+LO (green, dotted), NNLL + NLO (blue, dashed) and N$^3$LL + NNLO (red, solid) at $\sqrt{s} = 13~\mathrm {TeV}$ for the fiducial volume defined in the text. The lower panel shows the ratio to the NNLL + NLO result

**Fig. 2:**
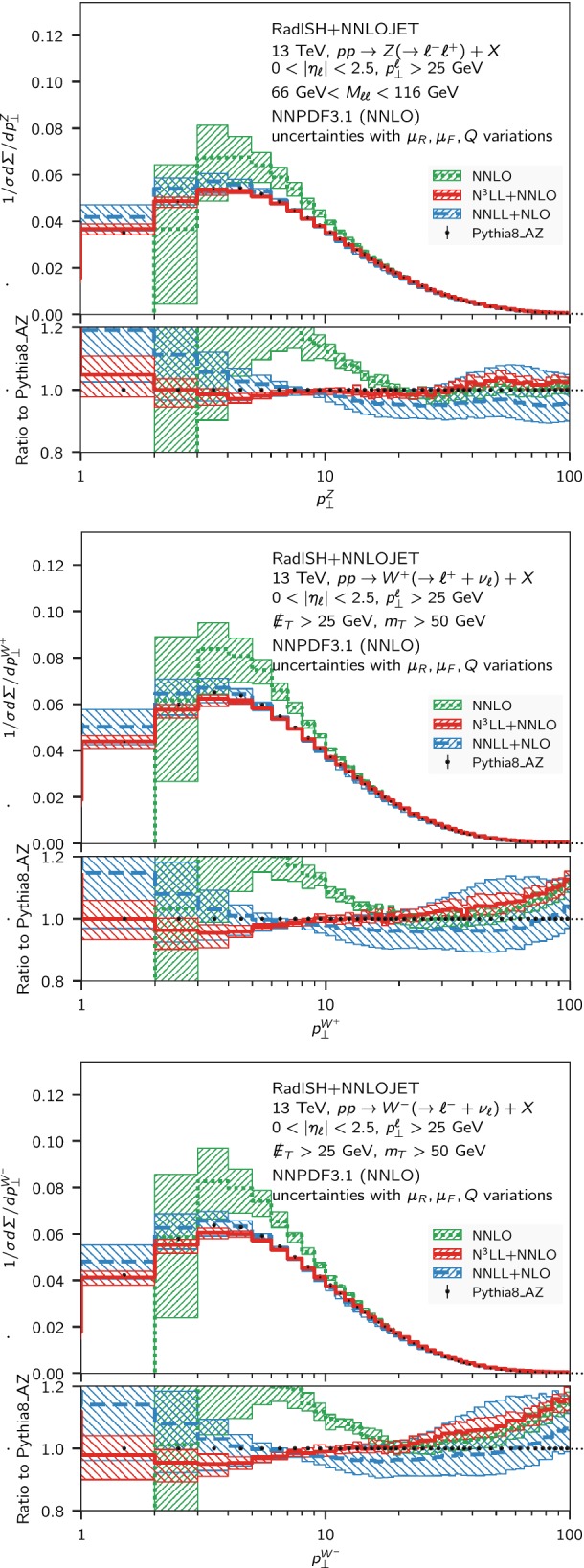
Comparison of the normalised transverse momentum distribution for neutral and charged Drell–Yan pair production at NNLO (green, dotted), NNLL + NLO (blue, dashed) and N$^3$LL + NNLO (red, solid) at $\sqrt{s} = 13~\mathrm {TeV}$ for the fiducial volume defined in the text. For reference, the Pythia8 prediction in the AZ tune is also shown, and the lower panel shows the ratio of each prediction to the Pythia8 result

### Fiducial distributions

We start by showing, in Fig. 1, the comparison of the *Z* and $W^{\pm }$ normalised distributions at NLL+LO (green), NNLL+NLO (blue), and N$^3$LL+NNLO (red) in the fiducial volumes defined above. The lower inset of each panel of Fig. 1 shows the ratio of all predictions to the previous state of the art (NNLL+NLO), with the same colour code as in the main panels. The difference between each prediction and the next order is of $\mathcal{O}(\alpha _s)$, both in the large $p_\perp$ region and in the limit $p_\perp \rightarrow 0$ where $\alpha _s L \sim 1$.

In comparison to the NNLL + NLO result, we note that the N$^3$LL + NNLO corrections lead to important distortions in the shape of the distributions, making the spectrum harder for $p_\perp > rsim 10~\mathrm {GeV}$, and softer below this scale. The perturbative errors are reduced by more than a factor of two across the whole $p_\perp$ range, and the leftover uncertainty is at the $5\%$ level. In general, we observe a good convergence of the perturbative description when the order is increased, although in some $p_\perp$ regions the N$^3$LL + NNLO and the NNLL + NLO bands overlap only marginally. This feature can be understood by noticing that, as mentioned in Sect. 2.2, both predictions are normalised to the same NNLO total cross section. Since at large $p_\perp$ the NNLO corrections lead to an increase in the spectrum of about $10\%$, by unitarity of the matching procedure (that preserves the total cross section) this must be balanced by an analogous decrease in the spectrum in the region governed by resummation, as indeed observed in Fig. 1. We stress, nevertheless, that the two orders are compatible within the quoted uncertainties.

In Fig. 2, we show the comparison among the NNLO (green), the NNLL + NLO (blue), and N$^3$LL+NNLO (red) predictions, where the bands are obtained as discussed above. Alongside these results, we also show the Monte Carlo predictions obtained using the Pythia8 generator [87] with the AZ tune [3], that has been obtained from the *Z*-boson $p_\perp$ distribution at $7~\mathrm {TeV}$. At $7~\mathrm {TeV}$ and $8~\mathrm {TeV}$ the above tune is known to correctly describe the *Z* transverse momentum spectrum within a few percent for $p_\perp \lesssim 50~\mathrm {GeV}$ [3], and it has been employed in the extraction of the *W*-boson mass by the ATLAS collaboration [13]. Although it is currently unknown how this tune performs at $13~\mathrm {TeV}$ in comparison to the data, we use the Pythia8 prediction for reference in the following plots. In particular, the lower inset of each panel of Fig. 2 shows the ratio of all predictions to Pythia8. We observe a reasonable agreement between the N$^3$LL + NNLO predictions and Pythia8 below $30~\mathrm {GeV}$, while it deteriorates for larger $p_\perp$ values. This feature is particularly visible in the case of $W^{\pm }$ production.

**Fig. 3:**
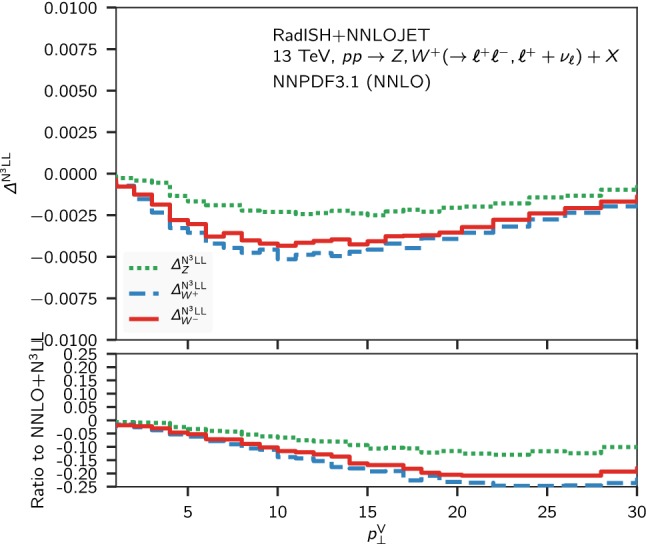
Difference Eq. (13) between the matched and the resummed predictions for the *Z* (green, dotted), $W^{+}$ (blue, dashed) and $W^{-}$ (red, solid) normalised distributions. The lower panel shows the ratio of $\varDelta ^\mathrm{N^3LL}$ to the N$^3$LL + NNLO matched result

A comparison of the N$^3$LL+NNLO band to the fixed-order one shows that the resummation starts making a significant difference for $p_\perp \lesssim 20~\mathrm {GeV}$, while above this scale the NNLO provides a reliable theoretical prediction. To further quantify the relative impact of the non-singular contributions in this region, we show in Fig. 3 the difference

13 $$\begin{aligned} \varDelta ^\mathrm{N^3LL}\equiv ( \; \mathrm {d} \Sigma ^\mathrm{N^3LL+NNLO}/\mathrm {d} p_\perp - \; \mathrm {d} \Sigma ^\mathrm{N^3LL}/\mathrm {d} p_\perp )/\sigma _\mathrm{tot}^\mathrm{NNLO} \end{aligned}$$

between the matched and the resummed predictions for the *Z* and $W^{\pm }$ normalised distributions. In the lower panel of the plot we show the relative size of $\varDelta ^\mathrm{N^3LL}$ with respect to the matched N$^3$LL + NNLO result, $\varDelta ^\mathrm{N^3LL}/( \Sigma ^\mathrm{N^3LL+NNLO}/\mathrm {d} p_\perp / \sigma _\mathrm{tot}^\mathrm{NNLO} )$. The non-singular contributions are somewhat larger for $W^{\pm }$; the relative size of $\varDelta ^\mathrm{N^3LL}$ with respect to the N$^3$LL+NNLO result is smaller than $5\%$ ($10\%$) for *Z* ($W^{\pm }$) for $p_\perp \lesssim 10$ GeV, and becomes larger than $10\%$ ($20\%$) for $p_\perp > 20$ GeV.

### Ratio of *Z* / *W* and $W^-/W^+$ distributions

Another set of important quantities of interest are the ratios of the above distributions, which play a central role in recent extractions of the *W*-boson mass at the LHC [13]. When taking ratios of perturbative quantities one has to decide how to combine the uncertainties in the numerator and denominator to obtain the final error.

One option is to try to identify the possible sources of correlation in the three processes considered here. From the point of view of the perturbative (massless) QCD description adopted in this study, one expects the structure of radiative corrections to such reactions to be nearly identical. This is certainly the case as far as resummation is concerned, since it is governed by the same anomalous dimensions and all-order structure in *W* and *Z* production. As a consequence, the resummation scale should be varied in a correlated manner in both predictions considered in the ratio. A similar argument can be made regarding the renormalisation scale $\mu _R$ and the factorisation scale $\mu _F$.

However, an important difference between *Z*, $W^+$, and $W^-$ production lies in the different combination of partonic channels probed by each process and, in particular, in the sensitivity to different heavy quark thresholds in the PDFs at small $p_\perp$. Therefore, it is not clear whether a fully correlated variation of the factorisation scale $\mu _F$ is physically justified. A more conservative uncertainty prescription is to vary the scales $\mu _R$ and *Q* in numerator and denominator in a fully correlated way, while varying $\mu _F$ in an uncorrelated manner within the constraint [36]

14 $$\begin{aligned} \frac{1}{2} \le \frac{x_{\mu _F}^\mathrm{num.}}{x_{\mu _F}^\mathrm{den.}} \le 2\,, \end{aligned}$$

where $x_{\mu _F}$ is the ratio of the factorisation scale to its central value. This corresponds to a total of 17 scale combinations.

**Fig. 4:**
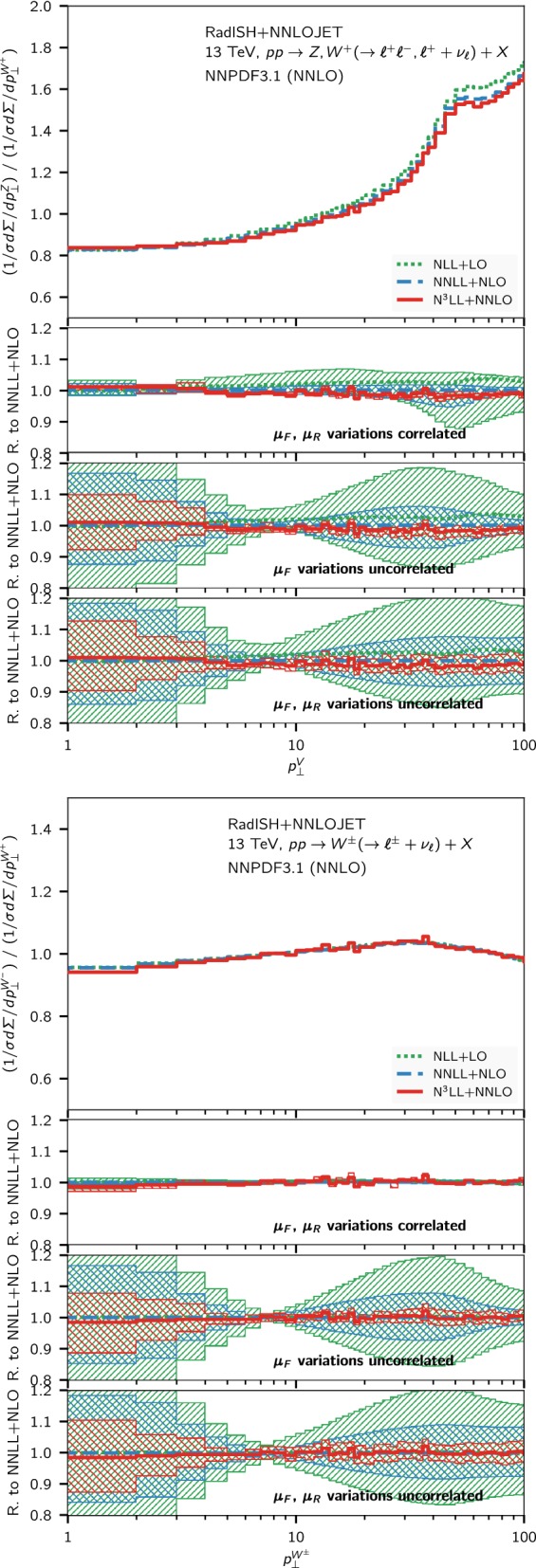
Ratios of $Z/W^+$ and $W^-/W^+$ normalised differential distributions at NLL + LO (green, dotted), NNLL+NLO (blue, dashed) and N$^3$LL + NNLO (red, solid) at $\sqrt{s} = 13~\mathrm {TeV}$. The three lower panels show three different prescriptions for the theory uncertainty, as described in the text

**Fig. 5:**
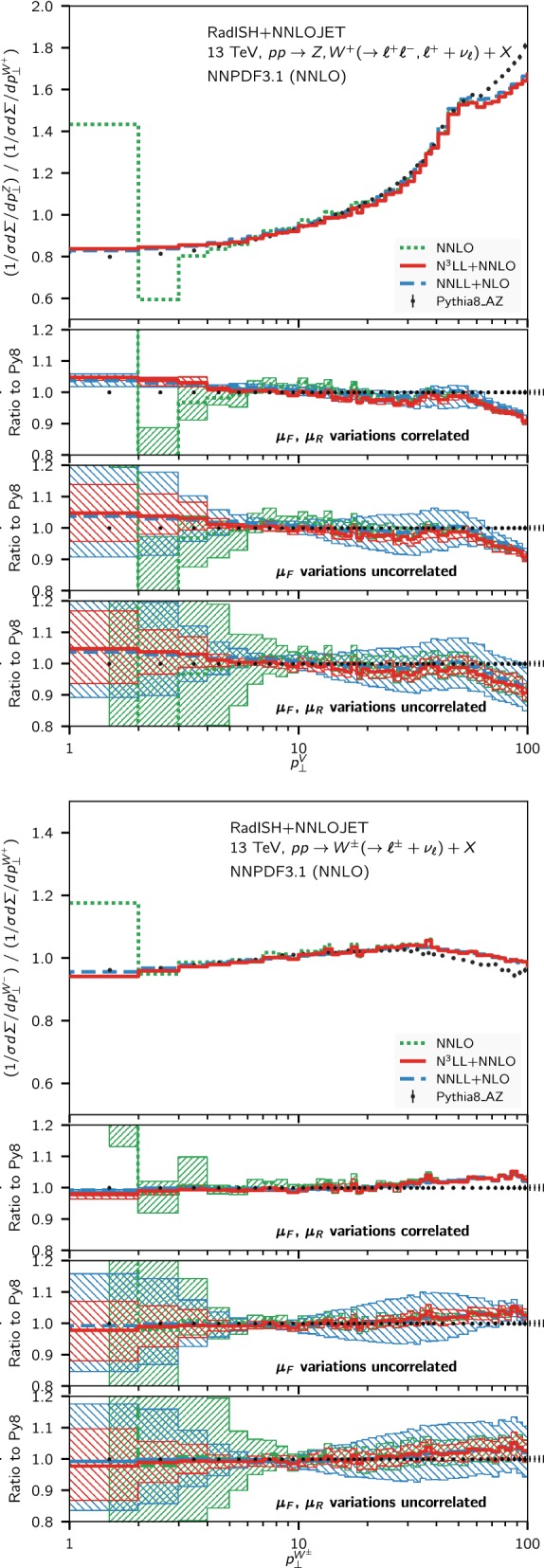
Ratios of $Z/W^+$ and $W^-/W^+$ normalised differential distributions at NNLO (green, dotted), NNLL+NLO (blue, dashed) and N$^3$LL + NNLO (red, solid) at $\sqrt{s} = 13~\mathrm {TeV}$. For reference, the Pythia8 prediction in the AZ tune is also shown, and the lower panels show the ratio of each prediction to the latter

Finally, for comparison we also consider the uncorrelated variation of $\mu _R$ and $\mu _F$ in the ratio, while imposing

15 $$\begin{aligned} \frac{1}{2} \le \frac{x_{\mu }^\mathrm{num.}}{x_{\mu }^\mathrm{den.}} \le 2\,, \end{aligned}$$

where $x_\mu$ is the ratio of the scale $\mu$ to its central value, with $\mu \equiv \{\mu _R, \mu _F\}$, together with a correlated variation of the resummation scale *Q*. This recipe amounts to taking the envelope of the predictions resulting from 33 different combinations of scales in the ratio.

To examine the reliability of the above uncertainty schemes, in Fig. 4 we analyse the convergence of the perturbative series for the ratios of distributions, by comparing the results at NLL + LO (green), NNLL + NLO (blue), and N$^3$LL + NNLO (red). The three lower panels in each plot show the theory uncertainties obtained according to the three prescriptions outlined above, respectively, in comparison to the old state-of-the-art prediction at NNLL + NLO. In the case of the $Z/W^+$ ratio (shown in the upper plot of Fig. 4), we observe that the different perturbative orders are very close to one another. The results are compatible even within the uncertainty bands obtained with the more aggressive error estimate, which in some bins is sensitive to minor statistical fluctuations due to the complexity of the NNLO calculation. This feature is strikingly evident in the case of the $W^-/W^+$ ratio (lower figure), where the excellent convergence of the series seems to indicate that either a fully correlated scale variation or the more conservative estimate of Eq. (14) is perfectly justified.

Figure 5 shows the comparison of the same two ratios ($Z/W^+$ and $W^-/W^+$) to the NNLO result (green), and to Pythia8. We observe that in both cases the N$^3$LL + NNLO calculation leads to an important reduction of the theory uncertainty. In particular, even with the most conservative estimate of the theory error, our best prediction leads to errors of the order of $5\%$, with the exception of the first bin where the perturbative uncertainty is at the $10\%$ level. The kink around $p_\perp \sim 50-60~\mathrm {GeV}$ in the $Z/W^+$ ratio (upper plot in Fig. 5) is due to the different fiducial selection cuts in the two processes. A change in the shape of the distributions around this scale is indeed visible in Fig. 2, at slightly different $p_\perp$ values for *Z* and $W^+$ production, respectively, that is reflected in the structure observed in Fig. 5. We find a good agreement between our best predictions at N$^3$LL + NNLO and the Pythia8 Monte Carlo in the small $p_\perp$ region of the ratios. However, the two predictions are not compatible within the quoted theory uncertainties if the scales are varied in a fully correlated manner. On the other hand, for $p_\perp > rsim 40~\mathrm {GeV}$, the Pythia8 result disagrees with the matched calculation. This behavior is not unexpected, since the nominal perturbative accuracy of Pythia8 is well below any of the matched calculations, and the AZ tune is optimised to describe the *Z* spectrum in the region $p_\perp \le 50~\mathrm {GeV}$ at $7~\mathrm {TeV}$.

## Conclusions

In this work, we computed the transverse momentum distributions of electroweak gauge bosons at the LHC to N$^3$LL + NNLO accuracy in QCD. This calculation opens up a new level of theoretical precision in the description of these observables. The new state-of-the-art prediction is obtained by combining the NNLO results from the NNLOjet program with the N$^3$LL resummation performed with RadISH. Our phenomenological study adopts fiducial selection cuts similar to the setup adopted by ATLAS in previous studies. The numerical results we presented are made available in electronic format as additional material alongside this manuscript.

We find that, in comparison to the fixed-order prediction, the resummation effects become important for $p_\perp \lesssim 20~\mathrm {GeV}$. The effect of the N$^3$LL+NNLO corrections with respect to the previous NNLL + NLO prediction is as large as $\sim 10\%$, and leads to significant shape distortions as well as to a substantial reduction in the perturbative uncertainty due to missing higher-order corrections. In particular, the distributions considered in this article are predicted with a residual uncertainty below the $5\%$ level across most of the $p_\perp$ spectrum. We also compared the results to the predictions obtained from the Pythia8 Monte Carlo with the AZ tune, that has been determined using the ATLAS experimental data for the *Z* boson transverse momentum at $7~\mathrm {TeV}$ [3].

Finally, we examined the ratios of the *Z* to $W^+$, and $W^-$ to $W^+$ distributions, which play an important role in the *W* mass extraction at the LHC. We consider different prescriptions for the estimate of perturbative uncertainties that rely on different degrees of correlation between the scales in the numerator and in the denominator. We find a remarkable convergence of the predictions for the ratios at different perturbative orders. This fact strongly indicates that the class of processes considered in this study feature very similar perturbative corrections suggesting that the perturbative sources of uncertainty are correlated to a large extent.

There are, however, additional sources of perturbative corrections to $W^{\pm }$ and *Z* production that we ignored in our study. In particular, at the level of the residual theoretical errors obtained in our predictions, PDF theory uncertainties [88, 89], QED corrections [90, 91], as well as a careful study of the impact of mass effects [92–102] become necessary. The correlation pattern between the uncertainties due to such effects may well be different from what we have observed in this paper, and a dedicated study must be performed in order to reliably combine these effects with the N$^3$LL+NNLO predictions presented here.

## Electronic supplementary material

Below is the link to the electronic supplementary material.

Supplementary material 1 (txt 41 KB)

## Data Availability

This manuscript has data included as electronic supplementary material.
